# Cellular Mediators of Inflammation: Tregs and T_H_17 Cells in Gastrointestinal Diseases

**DOI:** 10.1155/2009/132028

**Published:** 2010-02-08

**Authors:** Franco Pandolfi, Rossella Cianci, Danilo Pagliari, Raffaele Landolfi, Giovanni Cammarota

**Affiliations:** Institute of Internal Medicine, Catholic University of the Sacred Heart, Rome 00168, Italy

## Abstract

Human lymphocyte subpopulations were originally classified as T- and B-cells in the 70s. Later, with the development of monoclonal antibodies, it became possible to recognize, within the T-cells, functional populations: CD4^+^ and CD8^+^. These populations were usually referred to as “helper” and “suppressor” cells, respectively. However several investigations within the CD8 cells failed to detect a true suppressor activity. Therefore the term suppressor was neglected because it generated confusion. Much later, true suppressor activity was recognized in a subpopulation of CD4 cells characterized by high levels of CD25. The novel population is usually referred to as T regulatory cells (Tregs) and it is characterized by the expression of FoxP3. The heterogeneity of CD4 cells was further expanded by the recent description of a novel subpopulation characterized by production of IL-17. These cells are generally referred to as T_H_17. They contribute to regulate the overall immune response together with other cytokine-producing populations. Treg and T_H_17 cells are related because they could derive from a common progenitor, depending on the presence of certain cytokines. The purpose of this review is to summarize recent findings of the role of these novel populations in the field of human gastroenterological disease.

## 1. Introduction

In 1970 Gershon and Kondo described the role of T-lymphocytes in the induction of tolerance [1]. Much later true regulatory activity was recognized in a subpopulation of CD4^+^ cells characterized by high levels of CD25, the alpha-chain of IL-2 receptor [2]. This novel population is usually referred to as T regulatory cells (Tregs). IL-2 is essential for the generation of Tregs in the thymus and their survival, expansion, and suppressive function in the periphery [3]. 

The presence of alternative patterns of cytokine production has been well established in several pathological conditions and is usually referred to as T_H_1 cells that produce IL-2 and INF-gamma but not IL-4 and T_H_2 cells that produce IL-4 but not IL-2/IFN-gamma. Yet, the concept that the cells producing these alternative patterns of cytokines represent in humans irreversibly differentiated endpoints has been challenged [4, 5]. However, for the sake of simplicity, we will, as it is usually done, refer to these cells as T_H_1 and T_H_2 indicating not differentiated population but their pattern of cytokine production.

In this review we discuss experimental evidences derived from both human and mouse studies. For the sake of clarity, the reader should assume that we are reviewing human data unless differently specified.

## 2. Regulatory T-Cells (Tregs)

Regulatory T-cells constitute a minor subpopulation of CD4^+^ T-cell but their role is crucial for the control of autoreactive T-cells [6]. Naturally occurring CD4^+^CD25^+^ T-cells represent 5%–10% of peripheral CD4^+^ cells [7, 8]. They develop in the thymus from nonregulatory thymocytes during ontogeny [9]. Nevertheless not all the CD4^+^CD25^+^ T-cells can be considered Tregs. Most of the regulatory T-cells are CD4^+^CD25^high^ cells, which represent 2%-3% of the CD4^+^ T-cells [10]. However, recent data supports the idea that Tregs can also be CD25 negative [11, 12]. Regulatory T-cells are involved in the regulation of immune response, maintaining immunological self-tolerance and immune homeostasis [13], and the control of autoimmunity and cancer surveillance [14]. Therefore, Tregs play a key role in autoimmunity, allergy, cancer, infectious disease, and the induction of transplantation tolerance. As a consequence, abnormalities in the number and functions of Tregs have been implicated in the pathogenesis of the abovementioned clinical conditions [15].

Tregs are characterized by the expression of FoxP3 (Forkhead box P3), a transcriptional repression factor of the forkhead-winged helix family of transcription factors [16]. FoxP3 is physically associated with the Rel family transcription factors, nuclear factor of activated T-cells (NFATs) and NF-kappaB (NF-*κ*B), and blocks their ability to induce the endogenous expression of their target genes, including key cytokine genes [17]. Mutations in FoxP3 are associated with the inherited autoimmune disease, Scurfy in mice, and IPEX (immune dysregulation, polyendocrinopathy, enteropathy, and X-linked syndrome) in human [18]. IPEX is characterized by the presence of autoimmune disease in multiple endocrines organs, inflammatory bowel disease, allergies, and severe infections [16]. Beyond IPEX, mutations of FoxP3 have been seen associated also with an absence of Tregs [19]. 

A further evidence of the importance of FoxP3 derives from the observation by Hori et al. that retroviral gene transfer of FoxP3 converts naive T-cells into a regulatory T cell phenotype similar to that of naturally occurring CD4^+^ regulatory T-cells [20]. FoxP3 can also interact with the promoter of IL-127 receptor (IL-127R) and might contribute to reduce expression of CD127 in Tregs. Thus, CD127 expression inversely correlates with FoxP3 expression [21]. In 2008 Sakaguchi proposed FoxP3 as the crucial marker for Tregs development and function [14], and FoxP3 is currently one of the most accepted markers for Tregs. So Tregs are usually referred to phenotypically as CD3/CD4/CD25^high^/FoxP3^+^ cells. Recently, after the description of an inverse relationship between the expression of FoxP3 and CD127 [21], it has become possible to define an alternative phenotype of Tregs as CD3/CD4/CD25^high^/CD127^−^ cells.

In summary, FoxP3, IL-2, and CD25 are essential molecules for the development, function, and survival of Tregs. It is worth noting that induction of FoxP3 is also under the control of TCR and CD28 signaling. Moreover IL-2 and TGF-beta are important for the survival and proliferation of Treg cell precursors [22, 23]. 

A subset of human Tregs the FoxP3^+^ regulatory effector/memory-like T- (T_REM_) cells expresses CD39, nucleoside triphosphate diphosphohydrolase-1 [NTPDase 1], an ectoenzyme that degrades ATP to AMP. Different from mice, in which CD39 is expressed in all Tregs, in humans CD39 is a marker of a Treg subset likely involved in the control of the inflammatory autoimmune disease [24]. So, the expression of CD39 in concert with CD73, another ectonucleotidase present on the surface of lymphocytes, can further distinguish CD4^+^/CD25^+^/FoxP3^+^ Treg cells from other T-cells [25].

Alpha(E)beta(7)-integrin (CD103) is an integrin that enable the interaction with epithelial cadherin responsible for tissue-specific retention of lymphocytes. Thus, the integrin alpha(E)beta(7) can be regarded as a novel marker for subsets of functionally distinct Tregs specialized for crosstalk with epithelial environments [26]. CD103 has been seen also connected to the retention and homing of Tregs in inflamed tissues [27].

## 3. Tregs Subpopulations

Tregs are divided into two subpopulations, which are primarily defined by where they develop:

1. the natural Tregs (nTregs), which originate directly from the thymus, expressing at once high levels of CD25 and the transcriptional factor FoxP3,
2. the induced Tregs (iTregs), develop in the periphery from conventional CD4^+^ T-cells and whose expression can be modulated through antigenic stimulation under a variety of conditions.

A functionally important proportion of Tregs (the nTregs) originates from the thymus; nevertheless, in 2006, Chatenoud and Bach reported that Tregs are not present as such in the thymus; they derive from peripheral precursors that are CD4^+^CD25^−^ and differentiate into functional Tregs following adequate stimulation in the presence of the cognate antigen and specialized immunoregulatory cytokines, such as TGF-beta, IL-10, and IL-4; they are generally termed “adaptive” Tregs [28].

In addition several pathological and tolerogenic conditions, such as oral tolerance and the presence of retinoic acid, induce conversion of conventional T-cells into Treg cells. In particular, it has been shown that, in the presence of TGF-beta, all-*trans*-retinoic acid (ATRA) efficiently converts adult human peripheral blood naive CD4^+^ T-cells into Treg cells with stable and potent suppressive ability [29].

Moreover it has been demonstrated that a substantial percentage of Tregs had transient or unstable expression of the transcription factor FoxP3. These cells have been defined as “exFoxP3” T-cells, and they have an activated-memory T-cell phenotype and produce inflammatory cytokines. exFoxP3 cell numbers increase in inflamed tissues in autoimmune conditions. Analysis of the T-cell receptor repertoire suggested that exFoxP3 cells developed from both natural and adaptive Tregs [30].

## 4. Mechanisms of Action of Tregs

Several mechanisms have been proposed to explain the function of Tregs, but the effective mechanism of action of Tregs is not yet completely understood. Despite the numerous studies performed about Tregs functions, a number of fundamental questions concerning the origin, phenotypic nature, and mechanism of action of Tregs remain still elusive [15]. 

Currently it is believed that Tregs rely on several mechanisms as follows.

1. The first mechanism is metabolic disruption.
2. Tregs may regulate their target by modulating DC maturation via CD80/86 and CTLA-4 interactions.
3. Another mechanism is the cytolysis mediated by granzyme A or B in human and mice, respectively.
4. Moreover Tregs can produce inhibitory cytokines, such as IL-10, TGB-beta, and IL-35.
5. Finally Tregs are involved also in the regulation of peripheral tolerance to self-antigens.

Tregs were described initially as antigen nonspecific cells that act through an APC (Antigen Presenting Cell) independent mechanism. Tregs require activation via their TCR to become suppressive; but once activated, their suppressor effector function is completely nonspecific [31]. Nevertheless, subsequently it has been verified that may exist populations of Tregs that are antigen specific. More recently, a much larger analysis of hundreds of TCRs found little evidence that the Tregs population preferably recognized self-antigen; besides it was concluded that non-self-antigens are the cognate specificities of Tregs [32].

Other studies concluded that Tregs, like conventional T-cells, have a polyclonal TCR repertoire and are selected on self-peptides. Specificity for self-peptides directs the selection of CD4^+^CD25^+^ regulatory thymocytes by a process that is distinct from positive selection and deletion [33].

The growing number of inhibitory mechanisms ascribed to Tregs suggests that these cells take a multipronged approach to immune regulation. It is likely that the relative importance of each inhibitory mechanism is context dependent and modulated by the inflammatory milieu and the magnitude of the immune response. Taken together, these mechanisms provide a potent arsenal with which Tregs can act [34, 35].

### 4.1. Metabolic Disruption

Because of the presence of the alpha chain of IL-2 receptor (CD25), Tregs could be scavengers of IL-2, inhibiting the activation of other T-cells in the same environment, reducing the IL-2 availability. They do not produce IL-2, thus depend on IL-2 production by other cells. Reducing IL-2, Tregs induce apoptosis by IL-2 deprivation, which has been shown in several clinical conditions, including HIV disease [36].

Besides, these cells express also the transcriptional repression factor FoxP3 which is involved in the inhibition of cellular activity. So Tregs could consume IL-2 without activating their immune function and preventing, at the same time, the activation of other T-cells.

Moreover, because of the presence of the ectoenzymes CD39 and CD73 on the cellular surface of Tregs, these cells are also able to modulate cellular inhibition, through the production of pericellular adenosine (Ado) from ATP. ATP and Ado are important endogenous signaling molecules in immunity and inflammation. Extracellular ATP is a danger signal released by damaged cells; ATP behaves as chemoattractant for lymphocytes, activator of proinflammatory response, and inducer of local pain [37].

### 4.2. Cell-to-Cell Inhibition

One additional possibility to explain Tregs function is based on observation by Vignali [38], who referred that Tregs can mediate their inhibitory function by direct cell-to-cell contact. Wing et al. described the role of cytotoxic T-lymphocyte antigen-4 (CTLA-4), a protein highly expressed by Tregs, which inhibits the immune response [39]; CTLA-4 binds the costimulatory molecules CD80 (B7-1) and CD86 (B7-2) with higher affinity than CD28, inhibiting the second signal, necessary to immune activation [40]. Constitutive expression of CTLA-4 among CD4^+^ cells was restricted primarily to Tregs, suggesting that CTLA-4 expression by these cells is involved in their immune-suppressive function. These findings raise the possibility that Treg cell function contributes to the immune suppression characteristic of CTLA-4 signaling [41]. In vivo blockade of CTLA-4 for a limited period in normal mice leads to spontaneous development of chronic organ-specific autoimmune diseases, which are immunopathologically similar to human counterparts [42]. CTLA-4 is constitutively expressed on Tregs that express the transcriptional factor FoxP3. Furthermore, ectopic FoxP3 expression can phenotypically convert effector T-cells to regulatory T-cells [43]. Horwitz et al. revealed an important relationship between TGF-beta, CTLA-4, and FoxP3 in the generation of iTreg, but not nTreg [44]. 

Tregs also express the glucocorticoid-induced tumor necrosis factor receptor family-related gene (GITR, also known as TNFRSF18), a member of the tumor necrosis factor-nerve growth factor (TNF-NGF) receptor gene superfamily, which plays a key role in dominant immunological self-tolerance [45]. Stimulation of GITR abrogate CD4^+^CD25^+^ T-cell-mediated suppression and administration of a monoclonal antibody to GITR produce organ-specific autoimmune disease in otherwise normal mice [46]. 

One other cell surface molecule, Lymphocyte activation gene-3 (LAG-3), marks regulatory T-cell populations and contributes to their suppressor activity [47]. LAG-3 is a CD4-related transmembrane protein expressed by regulatory T-cells that binds MHC II on APCs. During Tregs-DCs interactions, LAG-3 engagement with MHC class II inhibits DC activation [48].

Another possibility of inhibition cell-to-cell contact-mediated of Tregs is based on the interactions, in lymphoid tissues, between Tregs and dendritic cells (DCs). In fact activated professional APCs (DCs, B-cells, and macrophages) can attract Tregs, through the secretion of common chemokines, such as CCL4, which is the most potent chemoattractan of Tregs [49]. Then, the cellular relation with DCs and Tregs helps to restrict DCs ability to form stable contacts with self-reactive T-cells, resulting in only transient interactions between DCs and naïve CD4^+^ T-cells [50].

### 4.3. Cytotoxic Activity

Tregs can suppress immune response also inducing apoptosis; this requires, in the target T-cells, the proapoptotic protein Bim, involved in the activation of caspases cascade [51]. Apoptotic mechanisms are used to regulate the development of thymocytes, the shaping of T cell repertoire, its selection, and the coordinate events leading to immune responses in the periphery [52]. Tregs can also control the immune response mediating the perforin/granzyme pathway; in fact Grossman et al. have shown that human adaptive Treg cells preferentially express granzyme B and can kill allogeneic target cells in a perforin-dependent manner. In humans, when Tregs are activated, they express granzyme A, but very little granzyme B [53]. This evidence shows that Tregs objectify their regulatory activity also through suppression by cytotoxic activity. 

Granzyme B is also important for the ability of NK cells and CD8^+^ T-cells to kill their targets. Recent data in the mouse has reported the important role of granzyme B in the suppression of immune system against tumor. In fact granzyme B has not been shown to be expressed in naive Treg cells but it is highly expressed in 5%–30% of Tregs in the tumor environment. Thus, in mouse, both granzyme B and perforin are relevant for Treg cell-mediated suppression of tumor clearance in vivo [54].

### 4.4. Inhibitory Soluble Factors

In contrast to these hypotheses of cell-contact suppression, there are quite a few reports indicating that Tregs could secrete several cytokines, as TGF-beta and IL-10, for mediating suppression reviewed in [55], limiting effector T-cells function, and inhibiting recruitment of inflammatory myeloid cells such as neutrophils, eosinophils, and monocytes [50]. For instance, IL-10 is required for the control of colitis and homeostatic maintenance of the T-cell number by Tregs review in [55]. However, the production of IL-10 and TGF-beta by Tregs has been challenged by others [56]. 

IL-35, an inhibitory cytokine of recent description, may be specifically produced by Tregs and is required for maximal suppressive activity [57].

### 4.5. Peripheral Tolerance

Moreover Tregs are also involved in the induction of peripheral tolerance to self-antigen. Tolerance to self-antigens is generated through two fundamental mechanisms, which included the elimination of self-reactive cells in the thymus during selection (central tolerance) and generation of a variety of peripheral regulatory cells to control self-reactive cells that escape the thymus (peripheral tolerance) [43]. So, at the peripheral mechanisms of tolerance in CD4^+^ T-cells, which included functional anergy and deletion [58], it is also possible to include the function of suppression by Tregs. Thus, the existence of Tregs that actively suppress the function of conventional T-cells is a key mechanism by which the immune system limits inappropriate or excessive response [15].

## 5. T_H_17 Cells

The heterogeneity of CD4 T-cells was further expanded by the recent description of the novel subpopulation T_H_17, characterized by potent proinflammatory properties. These cells, together with T-cells populations, which mainly produce a T_H_1 or T_H_2 pattern of cytokines, INF-gamma, and IL-4, respectively, regulate the overall immune response. Since their discovery, efforts have been made in characterizing human T_H_17 cells and the factors involved in their differentiation and in understanding the role that these cells play in inflammation, protective immunity, and autoimmune diseases [59].

CD4^+^ T-helper (T_H_17) cells are characterized by the production of IL-17A and IL-17F [60]. T_H_17 cells have recently been defined as a unique subset of proinflammatory helper T-cells whose development depends on signaling initiated by IL-6 and TGF-beta, autocrine activity of IL-21, activation of STAT3, and induction of the retinoic acid related orphan nuclear receptor RORgammat [61].

T-helper cells produced from the thymus develop into different differentiation program, controlled by cytokines produced in response to microbial products by innate immune cells. Each differentiation program, other than being characterized by unique cytokine profile, INF-gamma for T_H_1 cells and IL-4 for T_H_2, can also be identified by specific transcription factor, as pivotal regulator of the T-cells differentiation. These transcription factors include T-bet for T_H_1 cells, and GATA-3 for T_H_2 cells [62].

RORgammat does not function in isolation but coordinates the activity of a series of other essential transcription factor in guiding the differentiation of T_H_17 cells [62]. RORgammat induces transcription of the genes encoding IL-17 and the related cytokine IL-17F in naive CD4^+^ T-helper cells and is required for their expression in response to IL-6 and TGF-beta, the cytokines known to induce IL-17. TGF-beta together with IL-6 or IL-21 initiates the differentiation, while the IL-23 stabilizes the generation of T_H_17 cells [63].

Moreover, T_H_17 cells are also characterized by the surface expression of CCR6, IL-23R, IL-12Rbeta2, and CD161. They also can express the transcription factor T-bet in addition to RORgammat. 

The origin of T_H_17 cells is controversial: in human, T_H_17 cells originate from CD161^+^ naive CD4^+^ T-cells precursor, which constitutively express RORgammat and IL-23R, in response to the combined activity of IL-1beta and IL-23. These findings implicate CD161 as a novel surface marker for human T_H_17 cells and demonstrate the exclusive origin of these cells from a CD4^+^ CD161^+^ T-cell progenitor [64].

T_H_17 cells play a role in various human diseases associated with inflammation and destruction such as rheumatoid arthritis, psoriasis, Crohn's disease, and multiple sclerosis, where IL-17 can be seen as a therapeutic target. These diseases have in common the local chronic inflammatory reaction with production of inflammatory cytokines, leading to matrix destruction and defective repair [65]. Infectious disease mouse models indicate that T_H_17 cells mediate protection against extracellular pathogens [66]. T_H_17 may also contribute to tumor progression due to the role of their cytokines in inflammation and tissue repair [67].

## 6. Plasticity of CD**4^+^** T-cells: Connection with Tregs and T_H_17 Cells

The differentiation of T-cells into several differentiation lineages with distinct effector function is well established in the literature. However, after the investigation of other CD4^+^ T-cell populations, such as T_H_1 and T_H_2 cells, nTregs and iTregs, and T_H_17 cells, it is not more possible to enunciate with absolute certainty that these CD4^+^ T-cells subsets represent irreversibly differentiated endpoints [5]. The development into T_H_17 cells has not been considered to represent a terminal product of its developmental program [68]. Moreover, it appears that expression of FoxP3 by iTregs or IL-17 by T_H_17 cells may not be stable and that there is a great degree of flexibility in their differentiation options [5]. 

There is a close relation between Tregs and T_H_17 cells. While in mice these cells originate from a common precursor, in human it has been reported a developmental link between Tregs and T_H_17 cells, in which TGF-beta is essential for the generation of both cells [63]. TGF-beta orchestrates T_H_17 and Tregs differentiation in a concentration-depended manner [62]. At low concentration, TGF-beta synergizes with IL-6 and IL-21 to promote IL-23R expression, favoring T_H_17 cell differentiation. High concentration of TGF-beta represses IL-23R expression and favors FoxP3^+^ Treg cells [69]. Besides, there is also an inverse correlation with the transcription factors that identify each of these two T-cell subpopulations. In fact in the presence of TGF-beta, CD4^+^ T-cells express both RORgammat and FoxP3, but RORgammat function is antagonized by FoxP3. Rather in the presence of proinflammatory cytokines and low concentration of TGF-beta, RORgammat expression is further upregulated, whereas FoxP3 expression and function are inhibited. These evidences show the importance of cytokines environment in the differentiation of CD4^+^ T-cell subsets, depending upon the balance of expression of the transcriptional factors RORgammat and FoxP3.

Moreover, several recent publications have demonstrated the importance of retinoic acid in the plasticity between Tregs and T_H_17 cells. Retinoic acid has been demonstrated to be a key regulator of TGF-beta dependent immune responses, capable of inhibiting the IL-6-driven induction of proinflammatory T_H_17 cells and promoting anti-inflammatory Treg cell differentiation [70].

## 7. Tregs and T_*H*_17 Cells in Gastrointestinal Tumors

Tregs play also a crucial role in the modulation of the immune response to tumor. In particular Tregs are inversely correlated with prognosis of human neoplasias. An increase of their number is associated to a more high risk of incidence of tumors. Compared with healthy patients, patients with gastrointestinal malignancies had a higher proportion of CD4^+^ CD25^+^ T-cells in peripheral blood [71].

Most available studies are related to the number of Tregs in peripheral blood of patients with solid tumors. However the most critical point could be to evaluate the presence of Tregs at the site of tumor. It has been shown that many solid tumors are characterized by the infiltration of lymphocytes, and this presence of Tissue infiltrating lymphocytes (TILs). It is essential to determine the T-cell subsets involved in the immune response to understand the pathogenesis of immune diseases [72, 73].

Tregs have been associated with prevention of antitumor immunity and the evasion of efficient recognition of tumor antigens. TILs have been shown to possess a specificity to tumor-associated antigens (TAAs), which are often self-antigens. Analysis of the composition of the specific T-cell receptor (TCR) of TIL could thus provide information on the nature of the antigens recognized by TIL [74].

Many studies have revealed that Tregs are increased in several malignancies, such as esophageal cancer [75], gastric cancer [76], pancreatic cancer [77], and hepatocellular cancer (HCC) [78]. The increasing proportion of Tregs may be related to immunosuppression and tumor progression in patients with gastrointestinal malignancies. There are several evidences concerning the inverse correlation with the prevalence of Tregs and the prognosis of tumors. While it has been reported a direct connection with the increase of these cells and a poor prognosis in esophageal and gastric cancer, in pancreatic cancer and in HCC, there has already not been reported a significant difference by clinical stage in the prevalence of Tregs among patients with colorectal carcinoma [71].

Because of their recent discovery, the role of T_H_17 cells in tumor is poorly understood. T_H_17 cells increase in gastric cancer [79], colorectal cancer, and HCC [80].

The role of T_H_17 cells has emerged as instrumental in cancer pathogenesis, since IL-17 promotes tumor growth, whether IL-23, also produced by T_H_17, is considered a cancer-associated cytokine because it promotes tumor incidence and growth.

In addition, T_H_17 cells have recently been described to contribute to human tumor immunity by inducing T_H_1 cytokines and recruit effector cells to the tumor environment [81].

## 8. Esophageal and Gastric Cancers

The direct relationship between the prevalence of Tregs in PBMCs and in TILs and the clinical outcome in patients with esophageal and gastric cancers is shown through several clinical evidences. In fact patients with these cancers have higher proportions of Tregs compared with healthy patients. The population of Tregs in patient with advanced esophageal and gastric cancers was significantly larger than that in patients with early esophageal and gastric cancers, both in PBMCs and TILs [75, 82]. In addition patients with these tumors, who have higher levels of Tregs, show more advanced stages and poorer survival rates.

## 9. Pancreatic Adenocarcinoma

In pancreatic cancer high levels of Tregs have been found as a marker of bad prognosis. The prevalence on Tregs is significantly higher in the peripheral blood, in the tumor microenvironment, and even in the lymph nodes TILs in the lymphatic metastasis, compared with normal donors [83]. Recent publication describes the expression of FoxP3 in pancreatic ductal adenocarcinoma cells, providing evidence that this could be an important tumor escape mechanism [84]. 

## 10. Hepatocellular Carcinoma (HCC)

It has been demonstrated that lymphocytic infiltration of the cancerous tissue of liver is indicative of a better survival after surgical resection of the tumor [85]. This evidence confirms the importance of the TILs in the favorable outcome of tumor. However patients with HCC have increased number of Tregs in their peripheral blood and there are also comparable high numbers of Tregs in TILs of the tumor microenvironment. This datum suggests that the increase in frequency of Tregs may play a role in modulation of the immune response against HCC and could be important in design of immunotherapeutic approaches [78].

## 11. Tregs in Liver Transplant

The liver is a privileged organ with a lower incidence of rejection than other organs. Recent evidence has pointed out that some transplanted patients can achieve operational tolerance, allowing withdrawal of immunosupression. This event is associated with increased levels of Tregs [86]. Tregs have been shown to play a pivotal role in transplant tolerance [87]; regarding their function of suppressive cells, Tregs may play a role in the regulation of alloreactivity.

## 12. Inflammatory Bowel Disease as Result of Imbalance of Proinflammatory and Regulatory T-Cells Responses

Inflammatory bowel diseases (IBDs), comprising Crohn's Disease (CD) and Ulcerative Colitis (UC), are characterized by chronic idiopathic intestinal inflammation, resulting from predisposing genetic (genes encoding proteins relevant to both innate and adaptive immunity: NOD2, STAT3, and IL-23 receptor, etc.) and environmental factors (specific Toll-like receptors (TLRs) ligands and antigens derived from commensal bacteria) acting on immunoregulatory system. IBD may be result of an imbalance of proinflammatory and regulatory T-cells responses. 

Studies of Tregs in celiac disease are anecdotal. A recent work shows a higher percentage of circulating Tregs in patients with active disease than patients after dietary treatment; this experimental evidence suggests that Tregs could act in extinguishing the ongoing intestinal inflammation and the immune response to dietary gluten antigens [88]. In IBD, the inflammation is induced by many different cytokine-mediated pathways [89]. IL-23, IL-17, and the recently described IL-32 [90] have been linked to the pathogenesis of several inflammatory disorders, including IBD. 

Data available before the full characterization of T_H_17 cells implicated a T_H_1 cytokine profile in the pathogenesis of Crohn's disease. More recently, mouse models revealed that tissue inflammation does not develop in mice deficient in the IL-23p19 subunit, whereas inflammation can be found in IL-12p35 deficient mice, suggesting that these models are associated with T_H_17 responses [91].

Studies in humans are anecdotal, but, latterly an increased number of RORgammat expressing cells have been detected in the lamina propria of patients with Crohn's disease [92]. In addition, T_H_17 cells were isolated from inflamed lesions of Crohn's patients [93]. Subsequently, increasing interest in the pathogenesis of IBD has been attracted by the role of retinoic acid.

As Bai et al. put it [94], vitamin A and its metabolites, such as retinoic acid (RA), are active agents with a broad range of functions involving immune cell differentiation and maintaining immune homeostasis. For instance, vitamin A and its metabolites are capable of ameliorating various models of autoimmunity. 

Deficiency of vitamin A can lead to exacerbated experimental colitis, and supplementation of vitamin A results in amelioration of diarrhea.

Elias et al. have shown that all-trans retinoic acid (ATRA) and other agonists of the retinoic acid receptor alpha (RARalpha) inhibit the formation of T_H_17 cells and promote FoxP3 expression [95], while Benson et al. have reported that ATRA enhances Treg growth and differentiation [96].

The close link between IL-23 and IL-17 has been reported as IL-23/IL-17 axis. If an important role of IL-23 is emerging, the function of IL-17 in IBD remains still unclear. According to what was reviewed by O'Connor et al. [97], it has been verified that the absence of IL-17A or IL-17R in T-cells led to an accelerated and severe wasting disease accompanied by higher expression of genes encoding T_H_1-type of cytokines. IL-17A can also modulate the T_H_1 polarization in vitro in inhibitory sense, suppressing the induction of the transcriptional factor T-bet, the “master regulator” of T_H_1 differentiation of T-cells. This datum indicates that the presence of IL-17 within its receptor (IL-17R) is a favorable prognostic factor in the modulation of the pathogenesis of IBD, diminishing the activation of T-cells in T_H_1 sense.

These cytokines are produced by subsets of CD4^+^ T-helper (Th) cells: in contrast to UC that is considered more of a T_H_2 response and CD that is associated with T_H_1 and T_H_17 profile [98–100]. But new T-cell subclasses Tregs exist independently of T_H_1 and T_H_2 and play a central role in modulating IBD. In fact, there are new evidences that defects in Treg cell function may underlie IBD and discuss evidence that altered T-cell dependent responses to bacterial proteins may be central to its aetiology [101]. In IBD there is a hyper-reactive immune response in the gut wall directed against the commensal intestinal bacterial flora, and the CD4 T-cells dominate the adaptive immune response [102].

## 13. Tregs in IBD

Treg cells expressing FoxP3 and/or IL-10 have a role in the immune homeostasis of the gut [103]. 

It has been well demonstrated in experimental colitis in mice that Tregs not only prevent colitis but also block the progression and reverse the pathology, via IL-10 and Transforming Growth Factor (TGF)-beta-dependent and -independent mechanisms [103], and they suppress both T-cell-dependent colitis and intestinal bacterial inflammation [104]. Furthermore, helminthes have been demonstrated to protect against colonic inflammation, and Tregs were suggested to be responsible for this protective effect; in fact helminthes impede T_H_1 and T_H_2 responses generating an immunoregulatory environment via Treg, IL-10, and/or TGF-beta [105].

In humans, during active IBD, there is an inverse correlation between the frequency of peripheral Tregs and the severity of disease [106, 107], and there is an increased number of Tregs in the lamina propria, in mesenteric lymph nodes, and inflamed intestinal mucosa, compared to healthy controls [107, 108]. In addition, the frequency of Tregs decreases with treatment and correlates with clinical response [109]. It is possible that Tregs are recruited from peripheral blood to the inflammation's sites to regulate immune homeostasis [110].

In vitro functional analyses of Tregs from the peripheral blood or intestinal mucosa of IBD patients have shown that Tregs have normal cell-contact-dependent, cytokine-independent suppressive capacity, even against pathogenic T-effector cells derived from the inflamed mucosa [101]. In addition, Tregs derived from IBD inflamed mucosa suppress proliferation and cytokine production [111]. 

But, there are some difficulties to study mucosal Tregs because the markers used for Tregs from peripheral blood are not unique to Tregs from the lamina propria. Furthermore, nonsuppressive activated T effectors, increased in IBD mucosa, can express FoxP3 [112, 113]. Furthermore, functional in vitro assays are performed without the influence of the inflamed tissue, where diverse response to cytokines or costimulatory interactions may modify Tregs suppressive activity. For example, IL-23 inhibits the generation of colonic FoxP3^+^ Tregs [114–116]. 

In recent years, much interest has been attracted by TLR that are essential in the development of antimicrobial responses, by inducing the production of antimicrobial proteins and peptides by a number of cells in the gut epithelium. Activation of macrophages through TLR results in the secretion of inflammatory cytokines and chemokines, thus leading to development of inflammatory responses. 

In addition, decreased IL-10 production in T-cells from IBD patients or dendritic cells failure in inducing Tregs or presence of T effectors resistant to suppression can alter the Tregs suppressive activity in vitro [117]. 

Current IBD therapies have a role on the number and functions of Tregs. In children affected by CD, there is an increased number of colonic FoxP3^+^ cells after infliximab therapy [118]. In children, in fact, in contrast to adults, there are reduced numbers of colonic Tregs compared to healthy subjects. 

Probiotics stimulate the generation of Tregs that produce high levels of IL-10 and suppress the proliferation of effector T-cells in an IL-10-dependent manner [119]. 

Finally, recent evidence implicates T_H_17 cells in the pathogenesis of Crohn's disease [91].

## 14. T_H_17 in IBD

T_H_17 cells have been shown to play a role in the pathogenesis of autoimmune and inflammatory diseases, including IBD. Although Treg cells are effectively recruited at inflamed mucosa in IBD, it is possible that Treg cells may have proinflammatory effects through their ability to differentiate into T_H_17 in the presence of IL-6 and/or IL-23 at sites of inflammation [120].

T_H_17 associated cytokines (IL-17A, IL-17F, IL-21, IL-22) are elevated in bioptic specimens from IBD inflamed colon and in the sera during active disease. While IL-17 expression is not detectable in normal colon, it is readily detectable in CD colonic specimens, is related to disease severity, and is significantly lower in UC than in CD [121].

The role of IL-17A in colitis is controversial: it has been shown to be proinflammatory (due to the production of proinflammatory cytokines, as IL-6 and IL-8, and chemokines, as MCP-1).

IL-21, with TGF-beta, drives T_H_17 differentiation, contributing to the development of colitis. IL-22, derived from infiltrating T-cells and NK, is protective and induces antiapoptotic signaling pathways in the intestinal epithelial cells maintaining the integrity of the epithelial barrier [121].

Genome-wide association studies have linked CD to a number of IL-23 pathway genes, including the encoding gene for IL-23 Receptor. Similar associations in IL-23 pathway genes have been observed in UC. IL-23R is a key differentiation feature of CD4-T_H_17 effector cells that are critical in mediating antimicrobial defenses, is highly expressed by T_H_17 cells, and has a role in their maintenance. Several polymorphisms in the IL-23R gene locus were associated with either susceptibility or resistance to CD [122]. 

IL-23 is essential in mice for development of IBD and has the major proinflammatory role. In absence of IL-23, colonic levels of proinflammatory cytokines, as TNF-alpha, IFN-gamma, and IL-6, are reduced, while Tregs colonic population is greater. 

IL-23 plays an essential role in the induction of intestinal inflammation by innate immune mechanism. Its expression is highly increased in the inflamed intestine but not in systemic sites as those of the spleen and the liver [122].

The differentiation of inflammatory T_H_17 and suppressive Treg subsets is reciprocally regulated by relative concentrations of TGF-beta, with the concomitant presence of proinflammatory cytokines favoring T_H_17 differentiation, such as IL-6, or Tregs differentiation, such as retinoic acid. The expansion and survival of T_H_17 T-cells are subsequently mediated by IL-23 [121]. 

The homeostatic cytokine IL-7 is essential to the maintenance of colitogenic memory CD4 cells, critical to the maintenance of experimental colitis. 

In summary, the IL-23/IL-17 axis is linked to chronic intestinal inflammation and has a beneficial role in intestinal protection and homeostasis.

## 15. Conclusions

Tregs and T_H_17 cells are two important T-cell functionally subpopulations characterized by a specific pattern of cytokine production whose differentiation is modulated by the cytokine microenvironment that has been demonstrated to take part in the pathogenesis of autoimmune disease, cancer, and organs allograft.

So, if in tumor the increase of Tregs and T_H_17 cells is inversely correlated with a favorable prognosis, in the IBD and celiac disease there are different situations: while the increase of Tregs prevents the autoimmunity reaction, an amount due of T_H_17 cells promotes the outbreak of chronic inflammation. Finally an increase on the proportion of Tregs in the liver transplant correlates with a most favorable success of the transplant, preventing reject and also allowing to reduce the use of immune suppressives, reducing its numerous side effects. Further studies on the role of T_H_17 cells in allografts might be performed to make clear the effective negative role of these cells in the organs transplants. 

In summary, Tregs and T_H_17 cells seem to represent useful and effective mediators in inflammation, autoimmunity, cancer, and allograft rejection, and their serum and tissue levels can be used in diagnosis, prognosis, and monitoring of several diseases.
